# Dual cutoffs of estrogen receptor positivity define prognostic and predictive subgroups in breast cancer

**DOI:** 10.1016/j.isci.2025.114486

**Published:** 2025-12-20

**Authors:** Takashi Takeshita, Hirotaka Iwase, Rongrong Wu, Takashi Ishikawa, Li Yan, Kazuaki Takabe

**Affiliations:** 1 Department of Breast and Endocrine Surgery, Kumamoto City Hospital, Kumamoto, Japan; 2 Department of Breast Surgery and Oncology, Tokyo Medical University, Tokyo, Japan; 3 Breast Surgery, Department of Surgical Oncology, Roswell Park Comprehensive Cancer Center, Buffalo, NY, USA; 4 Department of Biostatistics and Bioinformatics, Roswell Park Comprehensive Cancer Center, Buffalo, NY, USA; 5 Department of Surgery, University at Buffalo Jacobs School of Medicine and Biomedical Sciences, the State University of New York, Buffalo, NY, USA; 6 Department of Surgery, Yokohama City University, Yokohama, Japan; 7 Department of Surgery, Niigata University Graduate School of Medical and Dental Sciences, Niigata, Japan; 8 Department of Breast Surgery, Fukushima Medical University, Fukushima, Japan

**Keywords:** health sciences

## Abstract

Estrogen receptor (ER) expression informs treatment decisions in breast cancer, and the percentage of ER-positive cells may further influence clinical behavior. We analyzed four HER2-negative cohorts by integrating immunohistochemistry-based ER quantification with transcriptomic and immune profiling. The proportion of ER-positive cells was the strongest predictor of recurrence. Recurrence risk peaked around 45% ER positivity and decreased beyond this point, supporting 50% as a meaningful prognostic cutoff. Tumors with ER ≥ 50% demonstrated favorable survival, whereas ER <50% showed molecular features similar to triple-negative disease, including proliferative and metabolic pathway activation and p53/DNA repair signaling. Immune profiling identified immune depletion in ER-high tumors, B cell and macrophage enrichment in ER-low tumors, and strong immune activation in triple-negative tumors. In neoadjuvant chemotherapy cohorts, lower ER percentages were associated with greater chemosensitivity, and 14% distinguished response likelihood. These results indicate that continuous ER expression provides prognostic and therapeutic insight beyond current threshold-based classification.

## Highlights

• ER ≥ 50% defines a prognostic threshold for breast cancer survival
• ER <50% tumors show intermediate biology and active proliferation/metabolism
• ER <14% optimally predicts response to neoadjuvant chemotherapy
• Dual ER cutoffs enable biology-informed classification and tailored therapy for ER-low BC

## Graphical abstract

**Figure:**
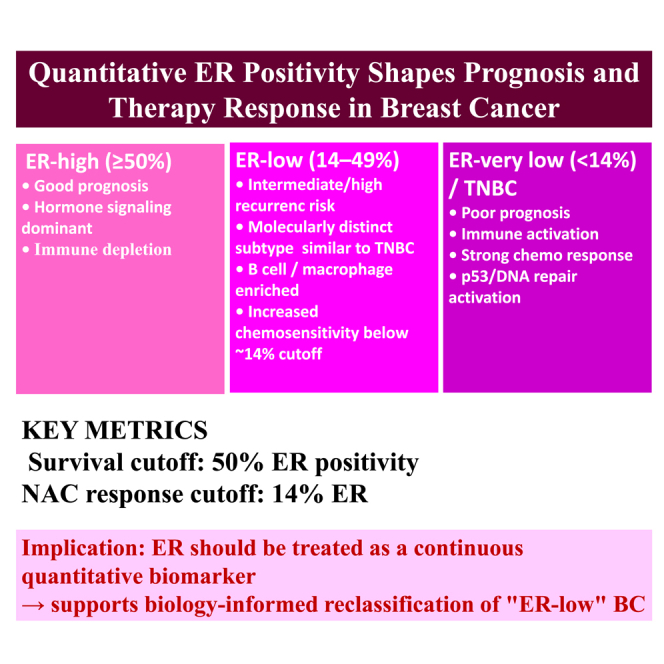

## Introduction

Breast cancer (BC) remains one of the most common cancers among women, with its incidence increasing by approximately 0.5% annually.^1^ Improving the prognosis of hormone receptor (HR)-positive BC, which accounts for 80% of all BC cases, is essential for enhancing overall BC survival rates.^2^ Over the past decade, research has concentrated on better stratifying patients at high risk of BC-related mortality.^3^ These efforts have primarily involved classifying BC patients based on distinct gene expression profiles and stratifying them according to clinical outcomes.^4^^,^^5^ Our group previously showed that high estrogen responsiveness is linked to specific gene pathways, immune cell suppression in the tumor microenvironment, and favorable prognosis, using early and late estrogen response scores calculated via gene set variation analysis (GSVA).^6^ Additionally, we utilized artificial intelligence on gene expression profiles to create a recurrence prediction model incorporating key BC treatment pathways and the tumor immune microenvironment.^7^

Immunohistochemical staining is a critical diagnostic tool for determining HR-positive status, with the American Society of Clinical Oncology and the College of American Pathologists (ASCO/CAP) guidelines recommending a 1% nuclear staining cutoff.^8^ However, the proportion of HR-positive cells is expected to significantly affect the efficacy of endocrine therapy (ET) and the risk of recurrence. As the percentage of HR-positive cells decreases, the cancer’s characteristics are expected to resemble those of triple-negative BC (TNBC).^9^ Despite years of debate regarding the optimal cutoff for HR-low BC, the specific percentage of HR-positive cells that correlates with a prognosis similar to TNBC has not been thoroughly investigated. Furthermore, clear guidelines for treating HR-low BC are lacking, complicating decisions on the appropriateness of adjuvant chemotherapy in such cases.

In this study, we aimed to test the hypothesis that machine learning could identify a clinically meaningful cutoff value for the proportion of estrogen receptor (ER)-positive cells, as quantified by immunohistochemistry (IHC), that influences prognostic outcomes or the efficacy of neoadjuvant chemotherapy (NAC). This cutoff was validated across BC cohorts from The Cancer Genome Atlas (TCGA) and the Gene Expression Omnibus (GEO), and further characterized by GSVA and tumor microenvironment profiling, with a particular focus on leukocyte infiltration.

## Results

### Identification and validation of a 50% ER-Positive cell cutoff as a prognostic factor in breast cancer recurrence

**Figure 1:**
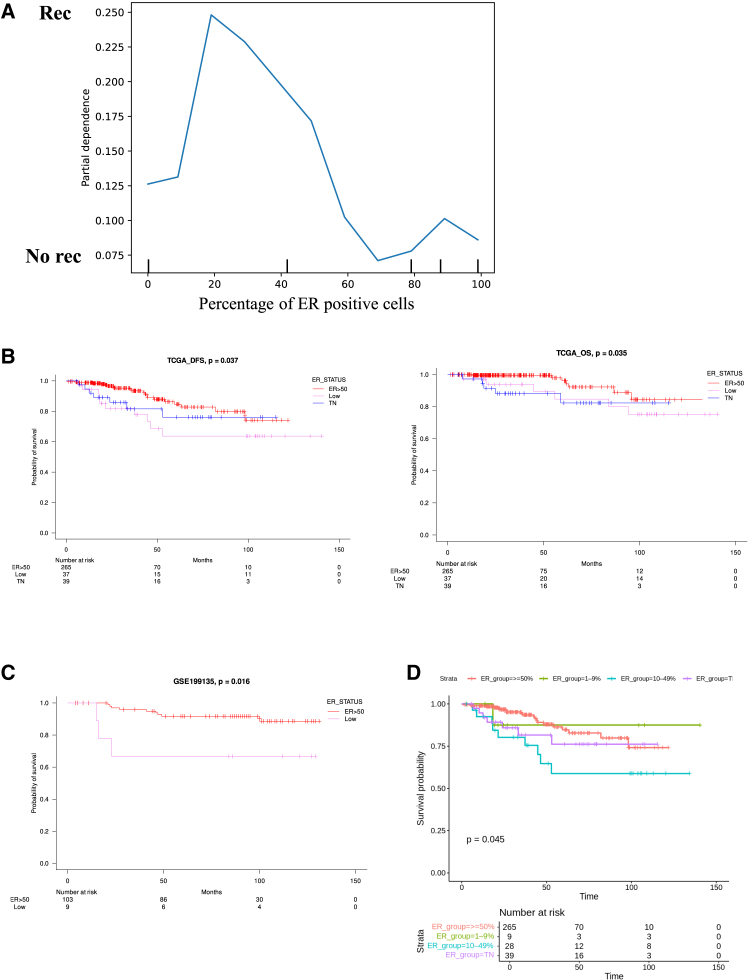
Clinical significance of IHC-based ER positivity thresholds in prognostic stratification of breast cancer (A) Partial dependence plots generated using the scikit-learn package, illustrating the influence of key features on recurrence prediction. The *y* axis indicates the partial dependence of recurrence. (B) Kaplan-Meier plots showing DFS and OS according to IHC-based ER positivity ≥50%, ER positivity <50%, and TN status in the TCGA cohort. Statistical comparisons were performed using log rank tests across the three groups (*N* = 341; DFS, *p* = 0.037; OS, *p* = 0.035). (C) Kaplan-Meier plots of RFS stratified by IHC-based ER positivity ≥50% and <50% in the validation cohort GSE199135. Statistical comparison was performed using a log rank test between the two groups (*N* = 112; *p* = 0.016). (D) Kaplan-Meier curves showing DFS in the TCGA cohort, further refining prognostic stratification by subdividing the IHC-based ER-positive group into 1%–9%, 10%–49%, and ≥50% categories alongside TN tumors. Statistical comparison was performed using a log rank test across the four groups (*N* = 341; *p* = 0.045). IHC, immunohistochemistry; ER, estrogen receptor; TN, triple negative; DFS, disease free survival; OS, overall survival; TCGA, The Cancer Genome Atlas; RFS, recurrence free survival.

We first sought to identify clinical features most predictive of recurrence. Using the scikit-learn package, the dataset was randomly divided into training and validation sets, and feature importance was assessed by permutation analysis (Table S1). The strongest predictors were the percentage of ER-positive cells (weight, 0.0395 ± 0.0237) and lymph node metastasis (weight: 0.0279 ± 0.0479). Whereas feature importance quantifies the relative contribution of variables, partial dependence plots illustrate their functional impact on recurrence prediction (Figure 1A). Recurrence risk increased as ER positivity rose from very low levels to approximately 20%–45%, but declined once ER positivity exceeded 45%. To formally determine the optimal threshold, survminersurv_cutpoint identified 50% as the cutoff that maximized discriminatory power for recurrence. Accordingly, tumors with ER positivity <50% were defined as “ER low” and those with ER positivity ≥50% were classified as “ER high”. This cutoff is consistent with the semiquantitative IHC scoring system, in which ER status is routinely reported as a percentage, thereby enhancing clinical applicability.

### Validation of the ER-Positive cell cutoff as a prognostic factor

The prognostic significance of the 50% cutoff was validated by Kaplan-Meier survival analysis and log rank testing in two independent cohorts (TCGA and GSE199135). In the TCGA cohort, patients were stratified into ER ≥ 50%, ER <50%, and TN groups. ER ≥ 50% was associated with significantly superior disease-free survival (*p* = 0.037) and overall survival (OS) (*p* = 0.033) compared with both ER <50% and TN (Figure 1B). Similarly, in an ER-positive/human epidermal growth factor receptor 2 (HER2)-negative cohort derived from our institutional dataset (GSE199135), patients with ER ≥ 50% had significantly improved recurrence-free survival (RFS) compared with those with ER <50% (*p* = 0.016) (Figure 1C). Consistently, permutation importance analysis in the GSE199135 cohort indicated that the percentage of ER-positive cells (importance = 0.0834) was the most influential predictor of recurrence, followed by menopausal status (0.0231), lymph node stage (0.0211), and age (0.0085), further supporting the prognostic relevance of the 50% ER cutoff (Table S1). Despite heterogeneity in treatment across cohorts, these findings consistently validate the prognostic value of a 50% cutoff. As only two cohorts were available, leave-one-cohort-out validation was not feasible. Instead, we employed a classical discovery-validation framework, with TCGA serving as the discovery cohort and GSE199135 as an independent external validation cohort. This design provides robust evidence supporting the generalizability of the 50% cutoff across distinct datasets.

To account for potential confounding factors that were not considered in the initial cutoff determination by the surv_cutpoint function, we performed additional multivariate Cox proportional hazards analyses in two independent cohorts (TCGA and GSE199135) (Table S2). The models included major clinicopathological variables such as age, tumor stage, lymph node stage, histology, and menopausal status as covariates.

In both cohorts, ER positivity ≥50% remained an independent favorable prognostic factor after adjustment for these confounders (TCGA: hazard ratio [HaR] = 0.35, 95% confidence interval [CI] 0.14–0.88, *p* = 0.03; GSE199135: HaR = 0.14, 95% CI 0.03–0.68, *p* = 0.01). The concordance indices of the multivariate models were 0.72 (TCGA) and 0.70 (GSE199135), indicating good discriminative performance. These results confirm that the 50% ER-positive cell threshold distinguishes prognosis independently of other clinicopathological characteristics.

The ASCO/CAP-recommended ER categories are defined as TN (<1% of tumor cell nuclei positive), ER low positive (1%–10% positive), and ER positive (>10% positive). However, Kaplan-Meier analyses have shown that tumors with ≥10% ER positivity often exhibit survival curves similar to, or even worse than, those with 1%–9% positivity, raising concerns about whether clinically meaningful low ER expression has been sufficiently captured.^9^^,^^10^^,^^11^ To further refine prognostic stratification, we subdivided the ER Positive group at the 50% threshold, thereby examining 1%–9%, 10%–49%, and ≥50% categories alongside TN tumors. This analysis revealed significant survival differences among the groups (log rank *p* = 0.045). Patients with ER ≥ 50% demonstrated the most favorable prognosis, those with ER 10%–49% had outcomes comparable to TN tumors, and ER 1%–9% tumors, although limited in number, showed an intermediate prognosis, better than TN but worse than ER ≥ 50% (Figure 1D). Collectively, these findings support both the biological and clinical validity of the 50% cutoff, while maintaining alignment with existing guideline-based categories.

### Distinct biological processes associated with low ER positivity

**Figure 2:**
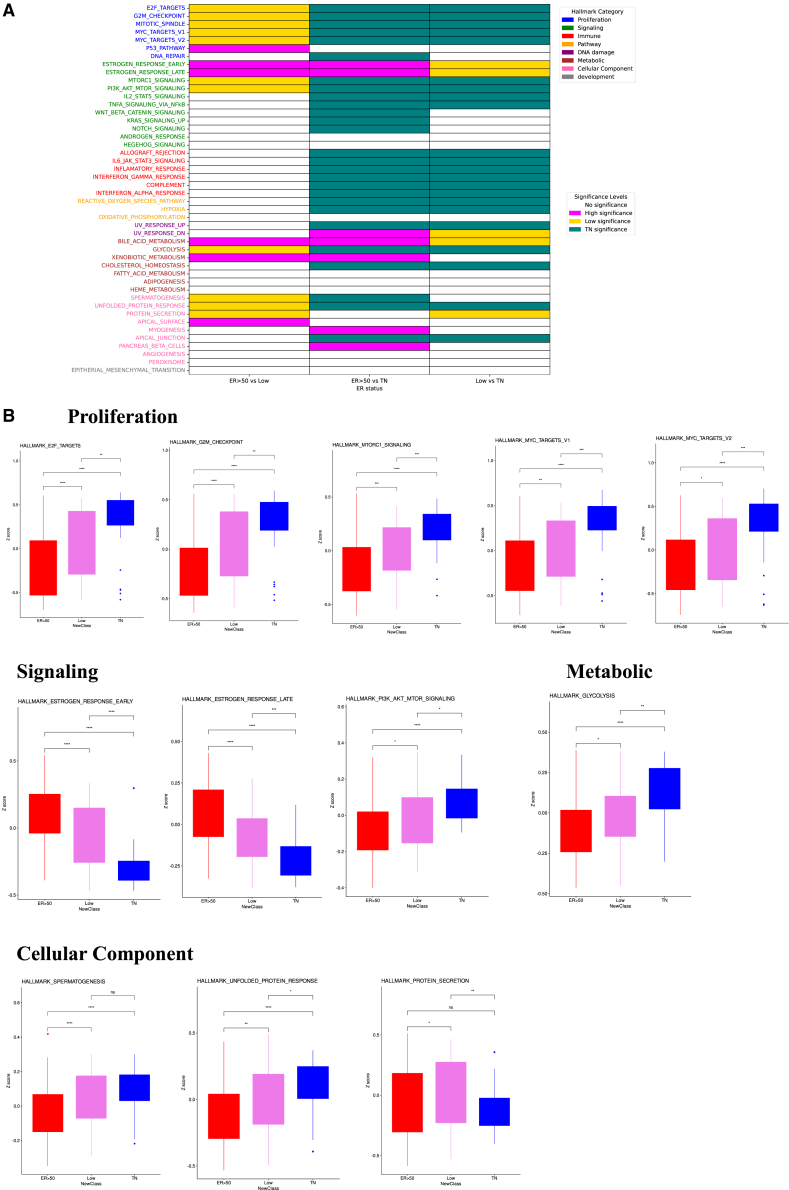
Distinct biological processes associated with low ER positivity by IHC in breast cancer (A) Heatmap illustrating the association of Hallmark gene set signatures with breast cancer subgroups defined by IHC-based ER ≥ 50%, ER <50%, and TN status in the TCGA cohort. (B) Boxplots showing that tumors with ER <50% exhibit distinct biological features compared with ER ≥ 50% and TN tumors, consistent with the differential enrichment patterns observed in the heatmap. Nonparametric Mann-Whitney U tests and contingency analyses were used to compare the three groups (*N* = 341). Data are presented as median with 25th–75th percentiles. ∗*p* < 0.05, ∗∗*p* < 0.01, ∗∗∗*p* < 0.001, ∗∗∗∗*p* < 0.0001. IHC, immunohistochemistry; ER, estrogen receptor; TN, tripe negative; TCGA, The Cancer Genome Atlas.

To explore biological mechanisms underlying low ER expression, GSVA was performed. The ER <50% group exhibited upregulation of proliferative pathways (E2F targets, G2M checkpoint, mitotic spindle, and MYC targets V1/V2) compared with ER ≥ 50%, although these were less pronounced than in TN tumors (Figure 2). Additional pathways enriched in ER <50% tumors included mTORC1 signaling, PI3K-AKT-mTOR signaling, glycolysis, and the unfolded protein response. Conversely, estrogen-responsive pathways (estrogen response early/late) remained more active than in TN tumors. Collectively, these results suggest that ER-low tumors represent a biologically distinct intermediate state between ER-high and TNBCs, characterized by proliferative activity and altered metabolic signaling. To further explore which specific pathways may dominantly influence the prognosis of ER <50% tumors, we performed Cox proportional hazards analyses using GSVA-derived pathway enrichment scores in the TCGA cohort (Table S3). The top-ranked pathways associated with poor prognosis included P53 signaling (HaR = 22.6, *p* = 0.085) and cell cycle-related pathways such as the G2M checkpoint and DNA repair, indicating that enhanced proliferative and genomic instability-related activity may contribute to unfavorable outcomes in this subgroup. Conversely, interferon alpha and gamma response pathways were associated with better prognosis (HaR = 0.30 and 0.28, respectively), suggesting a protective role of immune activation in ER-low tumors. In the GSE199135 cohort, the ER <50% group had very few recurrence or death events, leading to “too few events or samples” warnings during Cox modeling; thus, prognostic pathway analysis could not be statistically conducted in this dataset. This limitation likely reflects both the favorable outcomes and small sample size of this subgroup in the validation cohort. Collectively, these findings suggest that proliferative and DNA damage-related pathways (P53, G2M checkpoint) and immune-related responses (interferon signaling) may play key roles in determining prognosis among ER <50% tumors.

### Impact of ER positivity on chemotherapy efficacy and the immune microenvironment in NAC cohorts

#### Chemotherapy response and ER positivity

**Figure 3:**
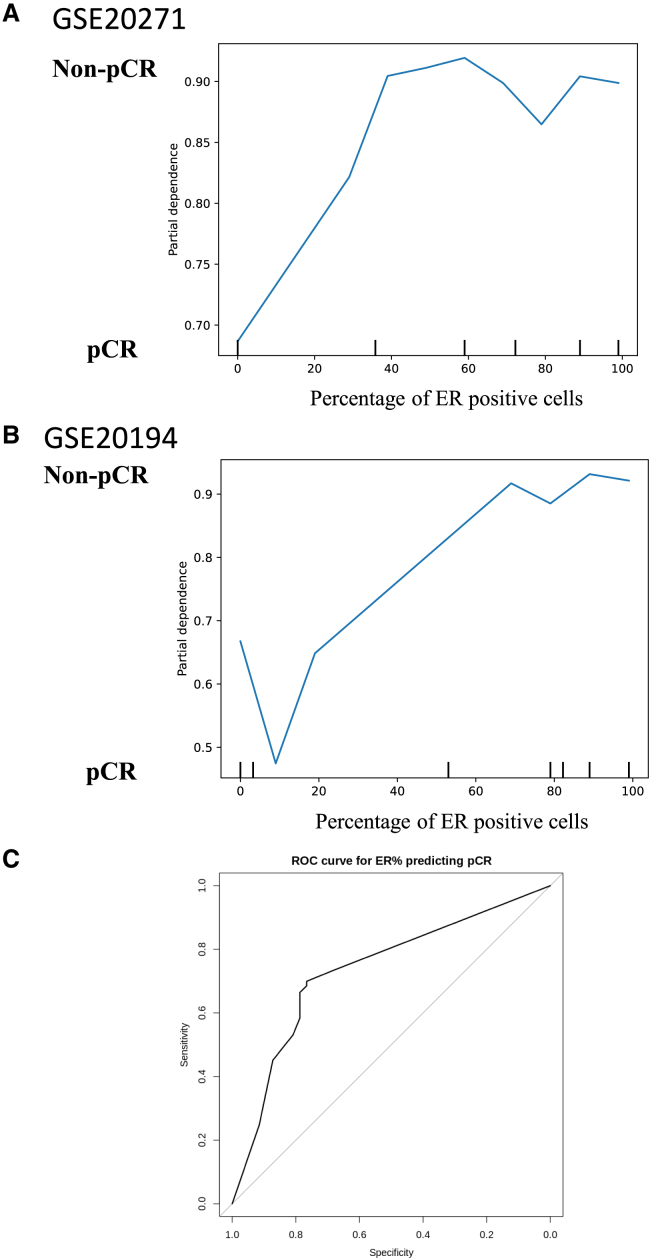
IHC-based ER positivity as a predictor of chemotherapy response in breast cancer (A) Partial dependence plot generated using the scikit-learn package in GSE20271, illustrating how key features, including IHC-based ER positivity, influence the probability of non-pCR. The *y* axis represents the partial dependence of non-pCR. (B) Partial dependence plot generated using the scikit-learn package in GSE20194, similarly demonstrating the impact of IHC-based ER positivity on non-pCR risk. (C) To integrate these observations across cohorts, ROC analysis was performed using the combined NAC datasets (GSE20271 and GSE20194), which identified 14% IHC-based ER positivity as the optimal cutoff for predicting chemotherapy response. IHC, immunohistochemistry; ER, estrogen receptor; pCR, pathological complete response; ROC, receiver operating characteristic; NAC, neoadjuvant chemotherapy.

We next investigated the impact of ER-positive cell expression on chemotherapy efficacy across two independent NAC cohorts, GSE20271 and GSE20194. The GSE20271 dataset was originally generated to explore candidate genes associated with non-pathological complete response (pCR) following neoadjuvant treatment with fluorouracil, doxorubicin, and cyclophosphamide, with or without weekly paclitaxel.^12^^,^^13^ In contrast, GSE20194, a component of the MicroArray Quality Control Consortium Project II, comprises tumor samples resected after six months of NAC, including regimens with paclitaxel, 5-fluorouracil, cyclophosphamide, and doxorubicin, thereby enabling the evaluation of intratumoral ER-positive cell proportion in relation to treatment response.^14^^,^^15^ In GSE20271, ER positivity and lymph node status emerged as the most influential predictors of non-pCR (Table S1). Partial dependence analysis demonstrated that the likelihood of non-pCR increased with ER positivity up to approximately 30%, after which it plateaued (Figure 3A). In GSE20194, ER positivity and tumor size were the key predictors of non-pCR (Table S1), with the frequency of non-pCR rising alongside ER positivity and peaking at around 60% (Figure 3B). To harmonize these observations across cohorts, receiver operating characteristic (ROC) analysis was performed using the combined NAC datasets, which identified 14% ER positivity as the optimal cutoff for predicting chemotherapy response (Figure 3C).

#### Pathway characteristics according to ER status

**Figure 4:**
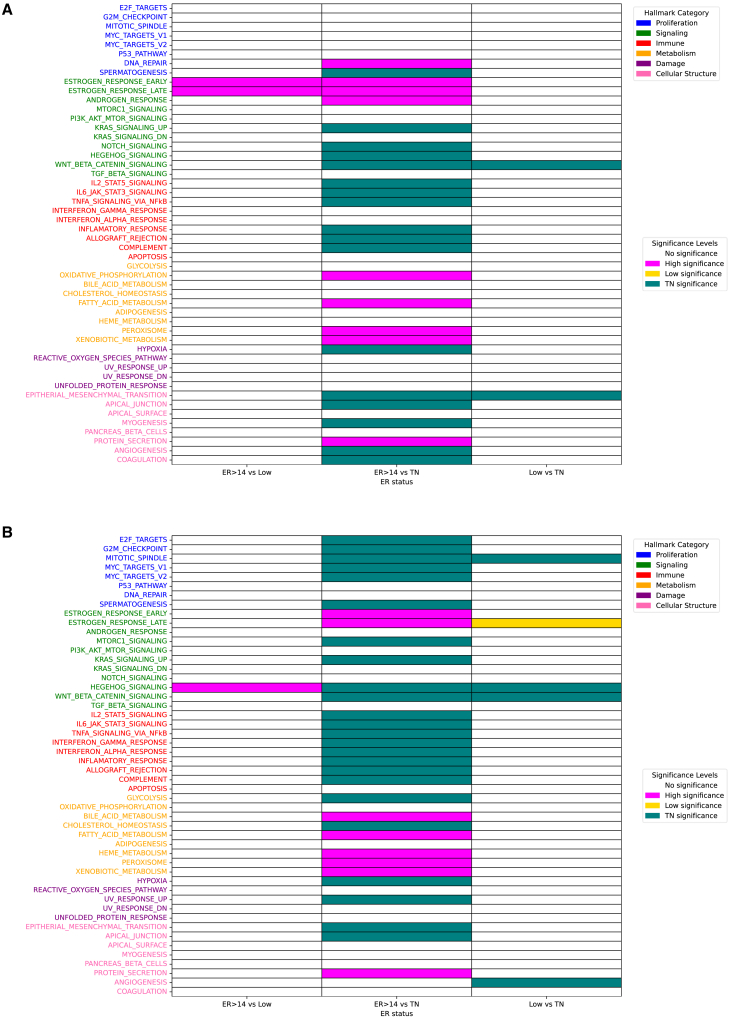
Association of hallmark biological processes with IHC-based ER positivity in NAC breast cancer cohorts (A) Heatmap showing the association of hallmark gene sets with tumors stratified by IHC-based ER positivity ≥14%, ER positivity <14%, and TN subgroups in GSE20271 cohort. (B) Heatmap showing the association of hallmark gene sets with tumors stratified by IHC-based ER positivity ≥14%, ER positivity <14%, and TN subgroups in GSE20194 cohort. Nonparametric Mann-Whitney U tests and contingency analyses were used to compare the three subgroups (GSE20271: *N* = 131; GSE20194: *N* = 203). IHC, immunohistochemistry; ER, estrogen receptor; NAC, neoadjuvant chemotherapy; TN, triple negative.

To investigate the biological context underlying these patterns, we performed pathway analysis using Hallmark gene sets. In GSE20271, High tumors were enriched in estrogen response early and late, highlighting strong hormonal dependency (Figure 4A). Compared with TN, they also demonstrated relative enrichment of DNA repair and androgen response. TN tumors again displayed broad activation of proliferative (spermatogenesis, KRAS, Hedgehog, WNT, and myogenesis), immune/inflammatory (IL6-JAK-STAT3, TNFα, allograft rejection, and complement), metabolic (oxidative phosphorylation, fatty acid metabolism, peroxisome, and xenobiotic metabolism), and stromal pathways (epithelial-mesenchymal transition [EMT], angiogenesis, and coagulation). Low tumors showed no clear enrichment beyond residual estrogen response, whereas TN tumors consistently exhibited a tumor-promoting microenvironment defined by angiogenesis, EMT, and stromal remodeling. In the GSE20194 cohort, High ER tumors showed minimal pathway activation, with only modest enrichment of Hedgehog signaling (Figure 4B). In contrast, TN tumors demonstrated broad activation of proliferative (E2F, G2M checkpoint, MYC targets, and mitotic spindle), metabolic (glycolysis, cholesterol homeostasis, and hypoxia), and immune/inflammatory pathways (IL6-JAK-STAT3, TNFα signaling, interferon α/γ response, inflammatory response, and complement). Oncogenic programs including Hedgehog, WNT, KRAS, and mTORC1 were also enriched, underscoring their aggressive biology. When compared with TN, low tumors retained residual estrogen signaling (Estrogen response late) but lacked activation of the proliferative and immune pathways that characterized TN tumors. Together, these analyses confirmed that TN tumors across both datasets were characterized by broad proliferative, metabolic, and immune/inflammatory activation. High ER tumors appeared comparatively quiescent with dominant estrogen signaling, while low ER tumors represented an intermediate group, retaining partial hormonal features without strong proliferative or immune activation.

#### Immune cell infiltration profiles

**Figure 5:**
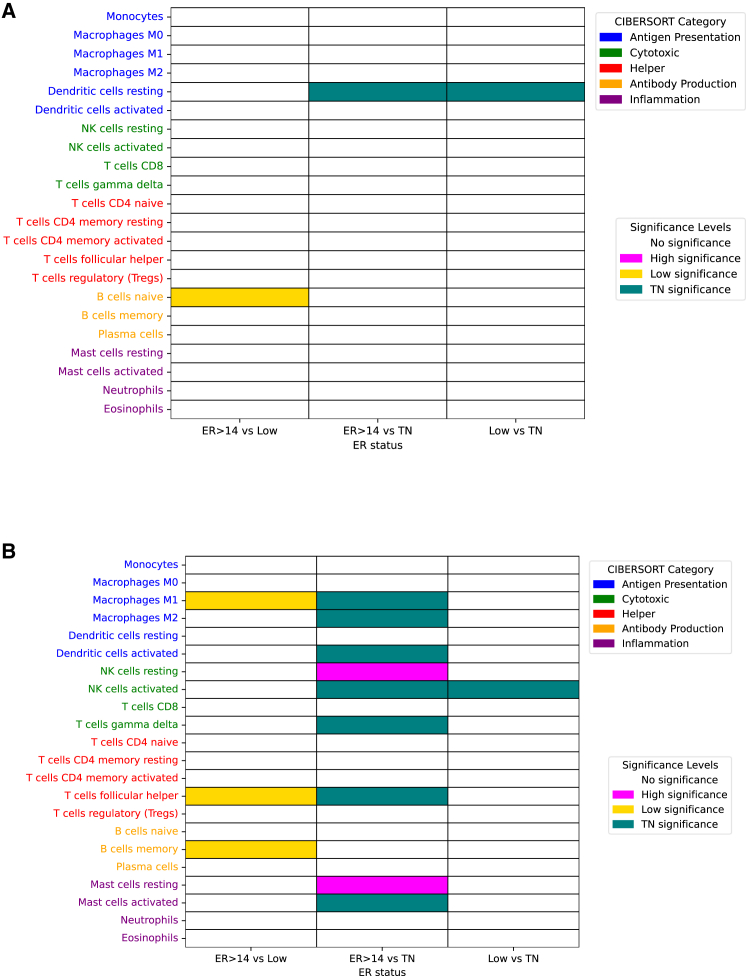
Association of intratumoral immune cell infiltration with IHC-based ER positivity in NAC breast cancer cohorts (A) Heatmap showing the estimated fractions of 22 intratumoral immune cell types using CIBERSORT in the GSE20271 cohort, with tumors stratified by IHC-based ER positivity (ER ≥ 14%, ER <14%) and TN status. (B) Heatmap showing the estimated fractions of 22 intratumoral immune cell types using CIBERSORT in the GSE20194 cohort, with tumors stratified by IHC-based ER positivity (ER ≥ 14%, ER <14%) and TN status. Nonparametric Mann-Whitney U tests and contingency analyses were used to compare the three subgroups (GSE20271: *N* = 131; GSE20194: *N* = 203). IHC, immunohistochemistry; ER, estrogen receptor; NAC, neoadjuvant chemotherapy; TN, triple negative.

We next assessed immune infiltration patterns across ER subgroups. In GSE20271, low tumors were enriched in naive B cells relative to High tumors, while TN tumors showed increased infiltration of resting dendritic cells compared with both high and low, suggesting enhanced antigen-presenting capacity. However, overall differences in immune infiltration were less pronounced than in GSE20194 (Figure 5A). In the GSE20194 cohort, low tumors showed increased infiltration of M1 macrophages, T follicular helper cells, and memory B cells compared with High tumors, suggesting enrichment of anti-tumor immune subsets. TN tumors exhibited the most immune-active phenotype, with broad enrichment of M1/M2 macrophages, activated dendritic cells, NK cells, γδ T cells, T follicular helper cells, and activated mast cells. Interestingly, no significant differences were observed between low and TN tumors, suggesting overlapping immune features (Figure 5B). Across both datasets, High ER tumors were consistently defined by low immune infiltration, low ER tumors demonstrated selective enrichment of B cell- and macrophage-associated subsets, and TN tumors exhibited broad and diverse immune activation, particularly evident in GSE20194. Because the immune microenvironment is a key determinant of response to NAC, CIBERSORT analysis was specifically performed on NAC-treated cohorts. Chemotherapy can substantially modulate tumor-immune interactions, and thus, evaluating immune cell composition in this context provides biologically and clinically meaningful insights into how ER expression influences immune remodeling and therapeutic response. In contrast, in primary BC cohorts such as TCGA and GSE199135, where patients had not received NAC, immune cell composition reflects baseline tumor immunity rather than therapy-induced modulation. Indeed, in TCGA and GSE199135, immune cell fractions showed minimal differences between ER-high and ER-low tumors, suggesting that ER-related immune distinctions become more evident only after chemotherapy exposure (Figure S1). These observations support the rationale for focusing the CIBERSORT analysis on NAC-treated cases to capture the dynamic immune-tumor interactions that are most relevant to treatment response and recurrence risk.

To further evaluate the potential impact of progesterone receptor (PR) expression on prognosis, we incorporated PR status into additional analyses using both the TCGA and GEO datasets (Tables S4 and S5). In the TCGA cohort (*N* = 341), PR negativity was observed in 11.7% of ER-high and 40.5% of ER-low tumors, showing a significant association between ER and PR expression (χ^2^ = 140.27, *p* = 3.48 × 10^−31^). However, when PR status was included in the multivariable Cox regression model together with age, menopausal status, tumor stage, lymph node stage, and histology, it was not independently associated with survival (HaR = 0.62, 95% CI 0.26–1.48, *p* = 0.28). Consistently, in the GEO datasets (GSE199135, GSE20271, and GSE20194), no significant or consistent associations between PR status and outcome were observed. These results indicate that, although ER and PR expression are correlated, PR status does not independently influence prognosis in ER-positive, HER2-negative BC.

## Discussion

In this study, we identified and validated two clinically meaningful thresholds of ER positivity that capture distinct aspects of BC biology and treatment response. For long-term outcomes, survival analyses consistently demonstrated that an ER-positive cell cutoff of 50% stratified prognosis most effectively, with ER ≥50% associated with superior survival, while ER <50% conferred outcomes more similar to TN tumors (Figure 1). To further validate the robustness of this threshold, we conducted multivariate Cox analyses in the primary BC cohorts (TCGA and GSE199135), which included major clinicopathological variables such as age, stage, histology, and menopausal status. Even after adjusting for these potential confounders, ER positivity ≥50% remained an independent favorable prognostic factor in both datasets (Table S2). These findings confirm that the prognostic value of the 50% cutoff is not merely attributable to differences in conventional clinicopathological features but rather reflects intrinsic tumor biology. The concordance indices of these multivariate models (0.72 for TCGA and 0.70 for GSE199135) further demonstrate their strong discriminative capacity. In contrast, the effect of NAC was most evident at a much lower threshold, with 14% emerging as the optimal cutoff for predicting pCR (Figure 3). These findings underscore the importance of considering ER expression not as a binary state but as a quantitative spectrum with differential clinical implications. The dual thresholds of 14% and 50% highlight distinct therapeutic vulnerabilities across the ER spectrum. Tumors with very low ER expression (<14%) behaved biologically like TNBC, demonstrating minimal dependence on estrogen signaling, limited responsiveness to ET, and marked chemosensitivity (Figure 4). Clinically, this suggests that patients in this group may benefit most from chemotherapy-based regimens and should be managed similarly to TNBC. Consistent with this observation, previous studies have shown that tumors with 1%–9% ER positivity closely resemble TNBC: Fujii et al. reported minimal benefit from adjuvant ET in this subgroup,^11^ and Iwamoto et al. demonstrated that most tumors with 1%–9% ER expression were molecularly similar to ER-negative, basal-like cancers.^9^ By contrast, tumors with intermediate ER expression (14%–49%) exhibited a hybrid phenotype, retaining partial estrogen responsiveness yet demonstrating enhanced proliferative and metabolic signaling, including activation of E2F, MYC, and PI3K-mTOR pathways (Figure 4). Notably, the higher GSVA scores for E2F_TARGETS and G2M_CHECKPOINT in the low ER group underscore its biological significance, particularly as cyclin-dependent kinase 4/6 inhibitors (CDK4/6i) directly target these pathways^16^ and have shown efficacy in HR-positive, HER2-negative MBC, as demonstrated in the PALOMA-3 trial.^17^^,^^18^ Similarly, elevated GSVA scores for PI3K_AKT_MTOR_SIGNALING in this group are clinically relevant, given that capivasertib, an AKT inhibitor, has demonstrated efficacy in the CAPItello-291 trial.^19^ Taken together, these findings suggest that treatment intensification, including the addition of chemotherapy, CDK4/6i, or novel targeted agents, may be required for patients with ER-low disease. In contrast, ER-high tumors (≥50%) were characterized by strong estrogen dependence, limited immune activation, and favorable long-term outcomes with ET, consistent with their established clinical behavior (Figure 2).

The NAC cohort analyses further reinforced these patterns. Increasing ER positivity was a strong predictor of non-pCR; the odds of non-pCR plateaued at moderate ER levels, consistent with the 14% threshold providing maximal discrimination for chemotherapy response (Figure 3). Across datasets, TN tumors showed broad activation of proliferative, inflammatory, metabolic, and stromal programs, aligning with their aggressive yet chemosensitive biology (Figure 4). ER-high (≥50%) tumors were relatively quiescent, whereas ER-low (<50%) tumors demonstrated an intermediate phenotype along this continuum. Within the ER-low spectrum, the <14% subgroup aligned most closely with TN features, while the 14%–49% subgroup retained residual estrogen signaling with attenuated, but detectable, proliferative and immune activity. Immune-infiltration analyses supported this gradient as follows: ER-high tumors were consistently immune-depleted, ER-low tumors showed selective enrichment of macrophage- and B-cell-associated populations, and TN tumors were the most immune-active, with broad enrichment of both innate and adaptive subsets (Figure 5). It is also important to note that these immune distinctions were predominantly observed in the NAC-treated cohorts (GSE20194 and GSE20271). Chemotherapy exposure is known to remodel the tumor immune microenvironment through mechanisms such as enhanced antigen presentation, immunogenic cell death, and cytokine induction, which can amplify subtype-specific immune differences. In contrast, in primary BC cohorts such as TCGA and GSE199135, where patients had not received NAC, CIBERSORT-derived immune cell fractions showed minimal differences between ER-high and ER-low tumors, reflecting baseline tumor immunity rather than treatment-induced modulation (Figure S1). These findings indicate that chemotherapy unmasked distinct immune phenotypes across ER subgroups, revealing that ER-low tumors exhibit selective activation of macrophage- and B cell-associated immune subsets under therapeutic pressure. Therefore, focusing the CIBERSORT analysis on NAC-treated cases provided biologically and clinically relevant insights into ER-dependent immune remodeling and its potential role in treatment response.

In large-scale studies that did not specifically interrogate low ER expression, Ali et al. modeled immune-cell proportions as quartiles to examine associations with survival and NAC response across BC subtypes.^20^ In ER-positive disease, memory B cells and monocytes were strongly associated with a lack of pCR; however, in survival analyses these same cell types correlated with better outcomes, highlighting a known discordance between pCR and long-term prognosis in ER-positive disease. Indeed, pCR after NAC is not generally accepted as a reliable surrogate endpoint for survival in ER-positive BC, as rising pCR rates have not consistently translated into improved outcomes.^21^ To further link NAC efficacy with survival determinants, we analyzed differentially expressed mRNAs in two published NAC cohorts.^22^ Among patients who achieved pCR, TP53, EGFR, CTNNB1, ERBB2, and HSPB1 emerged as candidate drivers of survival. In TN tumors, T-follicular helper cells were significantly associated with achieving pCR; memory B cells were likewise associated with pCR, suggesting a role for humoral immunity in mediating cytotoxic-therapy response, whereas M2 macrophages were associated with failure to achieve pCR and with chemotherapy resistance. Notably, the positive association of memory B cells with pCR in TN disease contrasts with their association with non-pCR in ER-positive disease reported by Ali et al., underscoring subtype-specific immune correlates of response.

To further explore whether PR expression contributes to prognosis beyond ER levels, we additionally assessed the impact of PR status in both the TCGA and GEO cohorts (Tables S4 and S5). Although PR negativity was more frequent among ER-low tumors, multivariate analyses adjusting for major clinicopathological factors demonstrated that PR status was not independently associated with survival. These findings suggest that, while ER and PR expression are correlated, the prognostic implications in ER-positive, HER2-negative BC are predominantly determined by the quantitative level of ER expression rather than by PR status itself.

Clinically, these findings carry several implications. First, the 50% cutoff provides a robust and guideline-concordant threshold for prognostic stratification, improving upon the binary ER-positive versus ER-negative classification. Second, the identification of 14% as the NAC response cutoff offers a complementary tool for predicting chemotherapy sensitivity, particularly relevant when considering NAC strategies in ER-low disease. Together, these thresholds suggest that ER-low tumors represent a heterogeneous and biologically distinct group that cannot be adequately captured by current classifications. ET alone is likely insufficient in these patients, and integration of chemotherapy or immune-based strategies should be considered. Finally, our results argue strongly for future clinical trials to evaluate ER-low BCs as a separate entity rather than grouping them with ER-high disease.

We establish ER ≥50% as a clinically relevant survival threshold, while identifying 14% as an optimal predictor of NAC response. These findings refine the biological and clinical characterization of ER-low BCs, positioning them as an intermediate and high-risk group distinct from both ER-high and TN tumors. Recognition of these dual thresholds supports a shift toward quantitative, biology-informed ER classification and highlights the need for tailored treatment strategies to improve outcomes in ER-low disease.

### Limitations of the study

The number of available cohorts was limited, precluding robust leave-one-cohort-out validation. A meta-analysis conducted by the Early Breast Cancer Trialists’ Collaborative Group demonstrated that tamoxifen conferred little to no therapeutic benefit in tumors with low ER expression.^23^ The near-universal administration of ET in GSE199135 (Table S6), coupled with the absence of an independent cohort incorporating IHC-based ER quantification and detailed ET data, substantially constrained our capacity to directly evaluate endocrine responsiveness. Prospective studies and preclinical models will therefore be essential to validate the therapeutic relevance of both the 50% survival cutoff and the 14% chemotherapy response cutoff.

## Resource availability

### Lead contact

Further information and requests for resources and reagents should be directed to and will be fulfilled by the lead contact, Takashi Takeshita (takashi.takeshita@hyper.ocn.ne.jp).

### Materials availability

This study did not generate new unique reagents.

### Data and code availability

• All data reported in this study will be shared by the lead contact upon reasonable request.
• This study did not generate original code.
• Any additional information required to reanalyze the data reported in this study is available from the lead contact upon request.

## Acknowledgments

K.T. is supported by the US National Institutes of Health grants R37CA248018, R01CA-250412, R01CA251545, R01EB029596, as well as the US Department of Défense BCRP grants W81XWH-19-1-0674 and W81XWH-19-1-0111.

## Author contributions

T.T.: writing – original draft, writing – review & editing, data curation, conceptualization, and formal analysis; H.I.: writing – review & editing; R.W.: formal analysis; T.I.: review & editing; L.Y.: formal analysis; K.T.: funding acquisition and writing – review & editing.

## Declaration of interests

T.I. received research grant from Chugai, Eisai, and Nippon-Kayaku, honoraria for lectures from Chugai, Pfizer, Astra Zeneca, Eli Lilly, Daiichi Sankyo, Kyowa Kirin, MSD, and Eisai and Board of Directors at Japanese Breast Cancer Society. The other authors have nothing to declare.

## STAR★Methods

### Key resources table

**Table:** 

REAGENT or RESOURCE	SOURCE	IDENTIFIER
**Deposited data**		

TCGA	TCGA PanCancer Atlas^24^^,^^25^ Liu et al.^26^ dataset	http://www.cbioportal.org/
GSE199135	Takeshita et al.^6^	https://www.ncbi.nlm.nih.gov/geo/
GSE20271	Tabchy et al.^12^ dataset	https://www.ncbi.nlm.nih.gov/geo/
GSE20194	Shi et al.^15^ dataset	https://www.ncbi.nlm.nih.gov/geo/

**Software and algorithms**		

Python 3.11.0	Python Software Foundation	https://www.python.org
Numpy v 1.23.4	Van Der Waltetal.^27^	https://numpy.org
SciPy v 1.9.3	Virtanen et al.^28^	https://scipy.org
Pandas v 1.5.1	pandas–Python Data Analysis Library	https://pandas.pydata.org
Seaborn v 0.12.1	Waskom^29^	https://seaborn.pydata.org
Matplotlib v 3.6.2	Hunter^30^	https://pypi.org/project/matplotlib/
R4.0.2	The R Foundation	https://www.r-project.org

### Experimental model and study participant details

We performed a retrospective analysis of four independent HER2-negative BC cohorts, comprising 341 women from TCGA and 446 women from GEO datasets (GSE199135, GSE20271, and GSE20194). Importantly, these cohorts included IHC-based quantification of ER staining, enabling direct evaluation of ER-positive cell proportions in relation to transcriptomic profiles and clinical outcomes (Table S6). Permutation importance identifies the variables that have the most significant impact on survival or pCR predictions, while partial dependence plots illustrate how these features influence those predictions.

### Method details

#### Gene set variation analysis (GSVA)

GSVA was performed using gene sets from the MSigDB Hallmark collection.^31^ The GSVA Bioconductor package (version 3.10) was employed to score the cancer hallmark gene sets, as previously described.^6^

#### CIBERSORT deconvolution algorithm

The relative proportions of 22 immune cell types within each tumor tissue were estimated using the CIBERSORT deconvolution algorithm.^32^ These cell fractions were calculated using the CIBERSORT online platform (https://cibersort.stanford.edu/), as demonstrated in previous studies^6^^,^^7^^,^^33^^,^^34^^,^^35^^,^^36^^,^^37^^,^^38^^,^^39^^,^^40^^,^^41^^,^^42^^,^^43^

#### Data analysis

Permutation importance and partial dependence plots were generated with the “scikit-learn” package. These analyses were conducted based on a Random Forest model implemented in the “scikit-learn” package to predict recurrence in BC based on clinical factors. Kaplan-Meier plots were created using the R packages “survival”, “Rcmdr”, and “survminer”.

### Quantification and statistical analysis

The chi-square test or Fisher’s exact test or the nonparametric Mann-Whitney U test and contingency analysis were used to assess baseline differences between binary variables. In the analysis of DFS, RFS and OS, the Kaplan–Meier method was used to estimate survival rates, and differences between survival curves were evaluated by the log rank test. Two-sided *p*-values <0.05 was considered as statistically significant for all tests.
