# Supplementary material for: Dual cutoffs of estrogen receptor positivity define prognostic and predictive subgroups in breast cancer
Source: iScience. 2025 Dec 20;29(1):114486. doi: 10.1016/j.isci.2025.114486 (PMC12804609; doi:10.1016/j.isci.2025.114486)
Supplement: Document S1. Figure S1 and Tables S1–S6 [file mmc1.pdf]

iScience, Volume *29*

Supplemental information

Dual cutoffs of estrogen receptor positivity define prognostic and predictive subgroups in breast cancer

Takashi Takeshita, Hirotaka Iwase, Rongrong Wu, Takashi Ishikawa, Li Yan, and Kazuaki Takabe

Supplementary data

Table S1. Permutation Importance Value of Clinical Features with the Greatest Impact on Recurrence Prediction in all cohorts

Cohort	Weight	Feature
TCGA	0.0395± 0.0237	Percentage of ER positive cells
	0.0279± 0.0479	Lymph node stage
	0.0047± 0.0114	Age
	0	Menopausal status
	0	Histology
	0	Tumor stage
GSE199135	0.083404	Percentage of ER positive cells
	0.023126	Menopausal status
	0.021088	Lymph node stage
	0.008453	Age
GSE20271	0.0667± 0.1111	Percentage of ER positive cells
	0.0667± 0.0594	Lymph node stage
	0.0121± 0.0822	Age
	0	Histology
	−0.0121± 0.0485	Tumor stage
GSE20194	0.0788± 0.0822	Percentage of ER positive cells
	0.0424± 0.0822	Tumor stage

	0	Lymph node stage
	−0.0303± 0	Histology
	−0.0545± 0.0804	Age

Abbreviations: TCGA, The Cancer Genome Atlas; GEO, Gene Expression Omnibus; ER, estrogen receptor.

Table S2 Multivariable Cox proportional hazards analysis of clinicopathological factors according to the percentage of ER-positive cells in the TCGA and GSE199135 cohorts

TCGA (N = 341)										
	**coef**	**Exp (coef)**	**Se (coef)**	**coef lower 95%**	**coef upper 95%**	**Exp (coef) lower 95%**	**Exp (coef) upper 95%**	**z**	**P-value**	**-log2(p)**
**Age**	0	1	0.02	-0.04	0.03	0.96	1.04	-0.19	0.85	0.23
**Tumor stage**	0.15	1.16	0.27	-0.38	0.68	0.69	1.98	0.56	0.58	0.8
**Lymph node stage**	0.63	1.88	0.17	0.3	0.96	1.35	2.61	3.75	<0.005*	12.45
**Histology**	0.06	1.06	0.27	-0.47	0.58	0.63	1.79	0.22	0.82	0.28
**Menopausal status**	0.47	1.6	0.53	-0.56	1.5	0.57	4.47	0.89	0.37	1.42
**ER High vs TN**	-0.23	0.79	0.55	-1.31	0.84	0.27	2.31	-0.43	0.67	0.58
**ER High vs Low**	-1.04	0.35	0.47	-1.96	-0.13	0.14	0.88	-2.23	0.03*	5.29

GSE199135 (N = 112)										
	**coef**	**Exp (coef)**	**Se (coef)**	**coef lower 95%**	**coef upper 95%**	**Exp (coef) lower 95%**	**Exp (coef) upper 95%**	**z**	**P-value**	**-log2(p)**
**Age**	-0.02	0.98	0.04	-0.09	0.06	0.91	1.06	-0.47	0.64	0.64
**Tumor stage**	Cases with many missing values were excluded from the analysis.									
**Lymph node stage**	0.89	2.44	0.69	-0.46	2.24	0.63	9.41	1.3	0.19	2.36
**Histology**	Cases with nearly identical histological types were excluded from the analysis.									
**Menopausal sstatus**	-1.31	0.27	1.04	-3.35	0.73	0.04	2.07	-1.26	0.21	2.27
**ER High vs Low**	-1.99	0.14	0.81	-3.58	-0.39	0.03	0.68	-2.44	0.01*	6.08

Abbreviations: TCGA, The Cancer Genome Atlas; GEO, Gene Expression Omnibus; ER, estrogen receptor; TN, triple negative. * indicates P-value < 0.05.

Table S3 Top 10 prognostic pathways identified by Cox proportional hazards analysis of GSVA scores in tumors with low ER-positive cell percentage (ER <50%, TCGA cohort, N = 37)

**TCGA (N = 37)**				
**Pathway**	**coef**	**HaR**	**P-value**	**-log2(p)**
**P53_PATHWAY**	3.117	22.574	0.085	3.561
**INTERFERON_ALPHA_RESPONSE**	-1.199	0.302	0.108	3.213
**INTERFERON_GAMMA_RESPONSE**	-1.280	0.278	0.108	3.205
**MYOGENESIS**	1.622	5.062	0.179	2.479
**IL6_JAK_STAT3_SIGNALING**	-1.127	0.324	0.199	2.326
**INFLAMMATORY_RESPONSE**	-1.119	0.327	0.210	2.254
**DNA_REPAIR**	1.727	5.624	0.210	2.253
**COMPLEMENT**	-1.449	0.235	0.212	2.240
**G2M_CHECKPOINT**	-1.033	0.356	0.228	2.132
**MITOTIC_SPINDLE**	-1.485	0.227	0.252	1.988

Abbreviations: TCGA, The Cancer Genome Atlas; HaR, hazard ratio.

Table S4 Distribution of PR status according to ER-positive cell percentage in all breast cancer cohorts

TCGA (N = 341)	PR status	
	Negative	Positive
ER-high ≥50%	31	234
ER-low <50%	15	22
TN	38	1
Chi-square test: $\chi2$=140.27, P = 3.479e-31*		

GSE199135 (N = 112)	PR status	
	Negative	Positive
ER-high ≥50%	18	85
ER-low <50%	2	7
Chi-square test: $\chi2$=0.00, P = 1.000e+00		

GSE20271 (N =130)	PR status	
	Negative	Positive
ER-high ≥14%	17	65
ER-low <14%	3	2
TN	38	5
$\chi2$ = 52.727, P = 3.552e-12*		

GSE20194 (N =132)	PR status	
	Negative	Positive
ER-high ≥14%	30	55
ER-low <14%	4	0
TN	36	7

$\chi 2 = 30.538$, df = 2, P = 2.337e-07*

Abbreviations: TCGA, The Cancer Genome Atlas; PR, progesterone receptor; GEO, Gene Expression Omnibus; ER, estrogen receptor; TN, triple negative.

* indicates P-value < 0.05.

Table S5 Multivariable Cox proportional hazards analysis of clinicopathological factors including PR status in TCGA breast cancer cohort and ER-low subgroup

TCGA ER low (N = 37)										
	coef	Exp (coef)	Se (coef)	coef lower 95%	coef upper 95%	Exp (coef) lower 95%	Exp (coef) upper 95%	z	P-value	-log2(p)
Age	-0.09	0.92	0.04	-0.17	-0.01	0.84	0.99	-2.13	0.03*	4.9
Lymph node stage	0.74	2.09	0.5	-0.24	1.71	0.79	5.54	1.48	0.14	2.86
Histology	-0.62	0.54	0.62	-1.84	0.6	0.16	1.83	-0.99	0.32	1.64
Menopausal status	2.51	12.28	1.24	0.07	4.94	1.08	140.08	2.02	0.04*	4.52
PR status	-0.91	0.4	0.76	-2.4	0.57	0.09	1.78	-1.2	0.23	2.13

TCGA (N = 341)										
	coef	Exp (coef)	Se (coef)	coef lower 95%	coef upper 95%	Exp (coef) lower 95%	Exp (coef) upper 95%	z	P-value	-log2(p)
Age	-0.01	0.99	0.02	-0.04	0.03	0.96	1.03	-0.29	0.77	0.37
Tumor stage	0.16	1.17	0.27	-0.38	0.69	0.69	2	0.58	0.56	0.83
Lymph node stage	0.64	1.91	0.17	0.32	0.97	1.37	2.65	3.84	<0.005 *	13
Histology	0.09	1.09	0.27	-0.43	0.61	0.65	1.84	0.34	0.73	0.45
Menopausal status	0.46	1.58	0.52	-0.56	1.48	0.57	4.38	0.88	0.38	1.41

**PR status**	**-0.49**	**0.62**	**0.45**	**-1.37**	**0.39**	**0.26**	**1.48**	**-1.08**	**0.28**	**1.84**
**ER High vs TN**	**0.07**	**1.07**	**0.6**	**-1.11**	**1.25**	**0.33**	**3.49**	**0.12**	**0.91**	**0.14**
**ER High vs Low**	**-0.65**	**0.52**	**0.58**	**-1.79**	**0.5**	**0.17**	**1.65**	**-1.11**	**0.27**	**1.89**

Abbreviations: PR, progesterone receptor; TCGA, The Cancer Genome Atlas.

* indicates P-value < 0.05.

Table S6. Patient and clinical characteristics of the corresponding TCGA and GEO cohorts

Clinical factor		Number of patients (%)			
		BC cohort		NAC BC cohort	
		TCGA	GSE199135	GSE20271	GSE20194
		(N = 341)	(N = 112)	(N = 131)	(N = 203)
Median Age(range)		59 (26-90)	60 (27-81)	49 (26-73)	50 (26-79)
Menopause state	Pre/Intermediate/Peri	108 (31.7)	34 (30.4)	NA	NA
	Post	233 (68.3)	78 (69.6)	NA	NA
Tumor stage	T1	91 (26.7)	50 (44.6)	9 (6.9)	18 (13.7)
	T2	197 (57.8)	20 (17.9)	61 (46.6)	117 (89.3)
	T3	52 (15.2)	2 (1.8)	26 (19.8)	32 (24.4)
	T4	1 (0.3)	0	33 (25.2)	34 (26)
Lymph node stage	N0	173 (50.7)	67 (59.8)	44 (33.6)	59 (45)
	N1	108 (31.7)	45 (40.2)	52 (39.7)	95 (72.5)
	N2	33 (9.7)	0	27 (20.6)	21 (16)
	N3	27 (7.9)	0	7 (5.3)	28 (21.4)
Histology	Ductal	212 (62.2)	110 (98.2)	122 (93.1)	126 (96.2)
	Lobular	92 (27)	1 (0.9)	8 (6.1)	4 (3.1)
	Others	37 (10.9)	1 (0.9)	1 (0.8)	2 (1.5)
Percentage of ER-positive cells	0	39 (11.4)	0	43 (32.8)	65 (49.6)

	1-9	9 (2.6)	0	5 (3.8)	9 (6.9)
	10-19	19 (5.6)	0	1 (0.8)	3 (2.3)
	20-29	3 (0.9)	2 (1.8)	3 (2.3)	4 (3.1)
	30-39	0	1 (0.9)	6 (4.6)	3 (2.3)
	40-49	6 (1.8)	6 (5.4)	6 (4.6)	6 (4.6)
	50-59	7 (2.1)	2 (1.8)	3 (2.3)	0
	60-69	9 (2.6)	3 (2.7)	9 (6.9)	7 (5.3)
	70-79	39 (11.4)	9 (8)	8 (6.1)	18 (13.7)
	80-89	19 (5.6)	21 (18.8)	20 (15.3)	40 (30.5)
	90-100	191 (56)	68 (60.7)	27 (20.6)	48 (36.6)
Adjuvant endocrine therapy	No		3 (2.7)		
	Yes		108 (96.4)		
Adjuvant chemotherapy	No		89 (79.5)		
	Yes		22 (19.6)		

Abbreviations: TCGA, The Cancer Genome Atlas; GEO, Gene Expression Omnibus; BC, breast cancer; NAC, neoadjuvant chemotherapy; NA, not applicable; ER, estrogen receptor.

Figure S1. Association of intratumoral immune cell infiltration with IHC-based ER positivity in TCGA and GSE199135 breast cancer cohorts.

A. Heatmap showing the estimated fractions of 22 intratumoral immune cell types generated using CIBERSORT in the TCGA cohort, with tumors stratified by IHC-based ER ≥50%, ER <50%, and triple-negative (TN) status. Nonparametric Mann–Whitney U tests and contingency analyses were used to compare the three subgroups (N = 341). **B.** Heatmap showing the estimated fractions of 22 intratumoral immune cell types generated using CIBERSORT in the GSE199135 cohort, with tumors stratified by IHC-based ER ≥50% and ER <50%. Nonparametric Mann–Whitney U tests and contingency analyses were used to compare the two subgroups (N = 112).

Abbreviations: IHC, immunohistochemistry; ER, estrogen receptor; TCGA, The Cancer Genome Atlas; TN, triple negative.

A

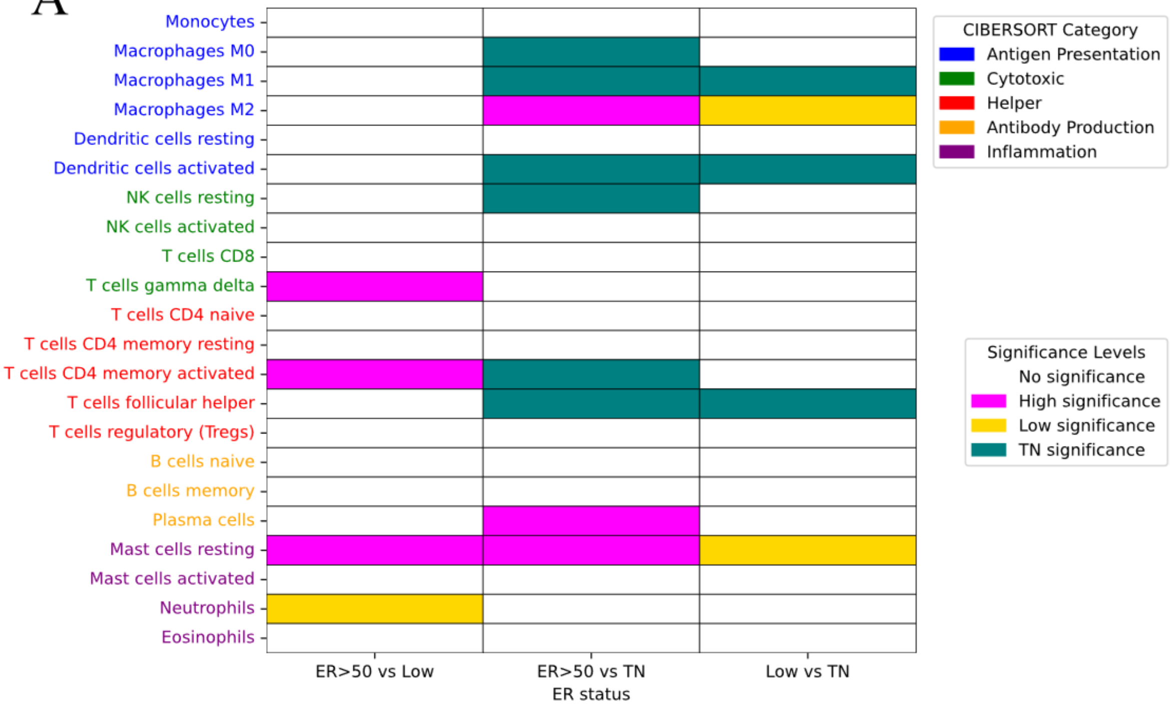
Monocytes
Macrophages M0
Macrophages M1
Macrophages M2
Dendritic cells resting
Dendritic cells activated
NK cells resting
NK cells activated
T cells CD8
T cells gamma delta
T cells CD4 naive
T cells CD4 memory resting
T cells CD4 memory activated
T cells follicular helper
T cells regulatory (Tregs)
B cells naive
B cells memory
Plasma cells
Mast cells resting
Mast cells activated
Neutrophils
Eosinophils
ER>50 vs Low
ER>50 vs TN
Low vs TN
ER status
CIBERSORT Category
Antigen Presentation
Cytotoxic
Helper
Antibody Production
Inflammation
Significance Levels
No significance
High significance
Low significance
TN significance

B

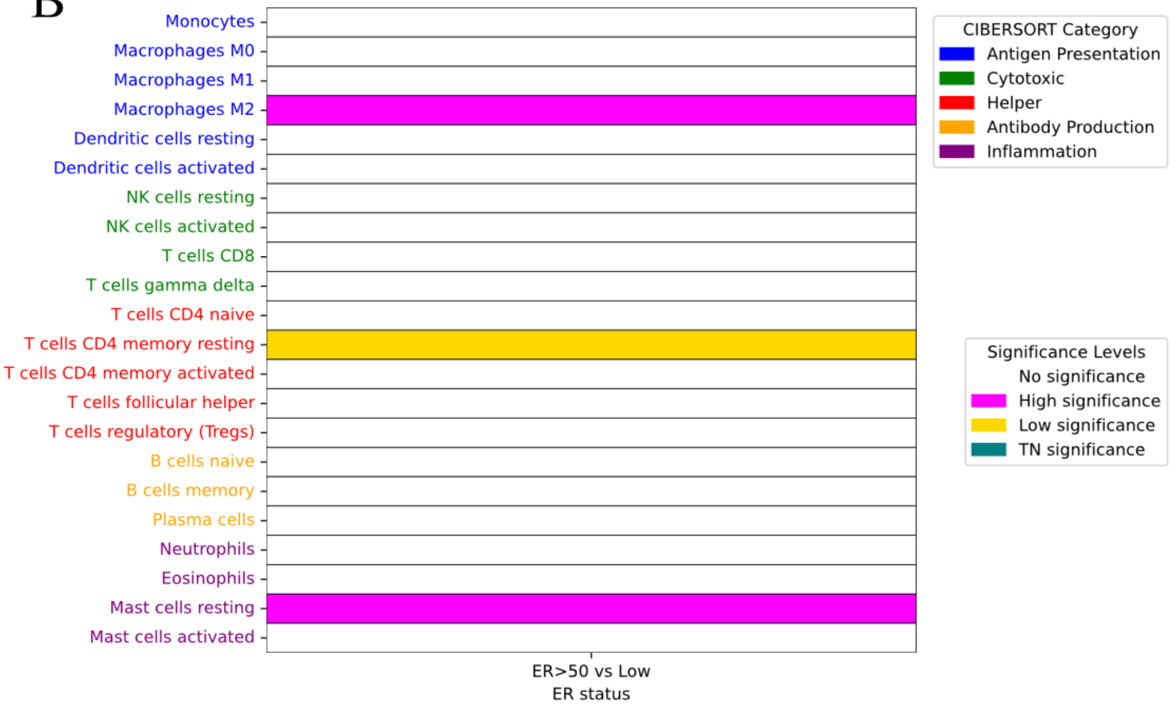
Monocytes
Macrophages M0
Macrophages M1
Macrophages M2
Dendritic cells resting
Dendritic cells activated
NK cells resting
NK cells activated
T cells CD8
T cells gamma delta
T cells CD4 naive
T cells CD4 memory resting
T cells CD4 memory activated
T cells follicular helper
T cells regulatory (Tregs)
B cells naive
B cells memory
Plasma cells
Neutrophils
Eosinophils
Mast cells resting
Mast cells activated
ER>50 vs Low
ER status
CIBERSORT Category
Antigen Presentation
Cytotoxic
Helper
Antibody Production
Inflammation
Significance Levels
No significance
High significance
Low significance
TN significance
